# Evaluation of Frailty Syndrome and Adherence to Recommendations in Elderly Patients with Hypertension

**DOI:** 10.3390/jcm10173771

**Published:** 2021-08-24

**Authors:** Piotr Pobrotyn, Aleksandra Pasieczna, Dorota Diakowska, Bartosz Uchmanowicz, Grzegorz Mazur, Mirosław Banasik, Aleksandra Kołtuniuk

**Affiliations:** 1 University Clinical Hospital in Wroclaw, 50-529 Wroclaw, Poland; Piotr@citodent.pl; 2 Department of Finance and Accounting, Kozminski University, 03-301 Warsaw, Poland; 1264-phdf@kozminski.edu.pl; 3 Department of Nervous System Diseases, Faculty of Health Sciences, Wroclaw Medical University, 51-618 Wroclaw, Poland; dorota.diakowska@umed.wroc.pl (D.D.); bartosz.uchmanowicz@umed.wroc.pl (B.U.); 4 Department of Internal Medicine, Occupational Diseases, Hypertension and Clinical Oncology, Wroclaw Medical University, 50-556 Wroclaw, Poland; grzegorz.mazur@umed.wroc.pl; 5 Department of Nephrology and Transplantation Medicine, Wroclaw Medical University, 50-556 Wroclaw, Poland; miroslaw.banasik@umed.wroc.pl

**Keywords:** hypertension, frailty syndrome, older adults, adherence, Tilburg Frailty Indicator, Hill–Bone Scale

## Abstract

Frailty syndrome (FS) often coexists with many diseases of the elderly, including arterial hypertension, and may affect the disease course and adherence to therapeutic recommendations. This study aimed to evaluate the relationship between frailty and adherence to therapeutic recommendations in elderly hypertensive patients. The study included 259 patients hospitalized between January 2019 and November 2020 due to exacerbation of hypertension symptoms. Medical records were used to obtain basic sociodemographic and clinical data. The study was based on the Tilburg Frailty Indicator (TFI) and the Hill–Bone Scale (HBCS). The obtained data were analyzed within a cross-sectional design. The mean frailty score indicated by the TFI questionnaire was 7.09 ± 3.73. The most prominent FS component was associated with the physical domain (4.24 ± 2.54). The mean overall adherence measured with the HBCS was 20.51 ± 3.72. The linear regression model testing the Hill–Bone “reduced sodium intake” score against the TFI domains showed no relationships between the variables. Another regression model for the Hill–Bone “appointment-keeping” subscale indicated significant predictors for physical and social TFI domains (*p* = 0.002 and *p* < 0.0001, respectively). For the Hill–Bone “taking antihypertensive drugs” variable, the regression model found significant relationships with all TFI domains: physical (*p* < 0.0001), psychological (*p* = 0.003) and social (*p* < 0.0001). Our study suggests that frailty in patients with arterial hypertension can negatively impact their adherence to therapeutic recommendations.

## 1. Introduction

In 2015, the number of patients with hypertension reached 1.13 billion globally, with more than 150 million cases in Central and Eastern Europe. The overall prevalence of hypertension in adults is 30–45% and, standardized by age: 20% and 24% in women and men, respectively [1,2,3].

There are two effective strategies for treating hypertension: lifestyle modification and treatment through pharmacotherapy. Lifestyle modification may decrease blood pressure. Furthermore, health-seeking behaviors also may reduce the risk of cardiovascular incidents; however, most patients still require pharmacotherapy despite nonpharmacological recommendation [1,4,5]. Effective lifestyle changes may be sufficient to delay or prevent the initiation of antihypertensive pharmacotherapy in patients with first-degree hypertension, and can also increase the effectiveness of ongoing drug therapy. The main limitation of nonpharmacological recommendations is their poor long-term adherence [6,7].

Recommended lifestyle changes that have demonstrated efficacy in lowering blood pressure include reduction in salt intake, restriction of alcohol intake, large vegetable and fruit intake, weight reduction and maintenance of desired weight, and regular physical activity [8,9]. Despite these recommendations, the treatment of hypertension based on lifestyle changes without drug therapy produces mixed results as reported elsewhere [9,10]. Therefore, as a difficult-to-manage condition, hypertension stands as a major risk factor for cardiovascular disease and mortality around the world [2,11,12,13]. Numerous studies have proven that inadequate adherence to therapeutic recommendations is the most important cause of poor control of hypertension [14,15,16,17]. Failure to follow recommendations for antihypertensive therapy is associated with an increased risk of cardiovascular incidents [18,19].

Frailty syndrome is a “physiological syndrome with a characteristic decrease in reserves and resistance to stressogenic factors resulting from the accumulation of decreased performance of various physiological systems, which consequently leads to susceptibility to the occurrence of certain consequences” [20]. Frailty is defined as a clinical syndrome in which three or more of the following criteria are present: unintentional weight loss (10 lbs in the past year), self-reported exhaustion, weakness (grip strength), slow walking speed, and low physical activity [21]. It is estimated that 13.4% of individuals over the age of 60 (incidence rate of 43.4 persons per 1000 persons) are affected by frailty syndrome [22]. It is more common in women, and its prevalence among the population increases in ageing societies [23], and upper-middle-income countries [24].

Frailty syndrome is an important issue in elderly patients that requires specialized diagnostics as well as primary and secondary prevention. It coexists with many diseases of old age, including hypertension, and can adversely affect the course of the disease and adherence to therapeutic recommendations. In addition, frail older patients have a poorer quality of life, decreased mood, and cognitive impairments [25].

This study aimed to assess the impact of frailty syndrome on compliance with therapeutic recommendations in elderly patients with hypertension.

## 2. Materials and Methods

### 2.1. Participants and Settings

The research material involved 259 patients hospitalized from January 2019 to November 2020 in the Clinic of Angiology, Hypertension and Diabetology of the University Clinical Hospital in Wroclaw due to exacerbation of symptoms of hypertension.

The criteria for inclusion in the study included: hypertension diagnosed based on the Polish Society of Hypertension guidelines; prior history of hypertension for at least one-year before the study entry; age ≥ 65; hypertension treated pharmacologically with at least one antihypertensive drug; and patient consent to participate in the study. Criteria for exclusion from the study: hypertension lasting less than 12 months; age ≤65 years; hypertension treated only with nonpharmacological methods; no patient consent to participate in the study.

### 2.2. Ethical Considerations

The research project was approved by the Bioethics Committee of Wroclaw Medical University, Poland (permission no. KB–114/2016). Participation in the study was anonymous and voluntary. All patients were informed about the study, and their written consent to participate in the study was required. The study was carried out following the Declaration of Helsinki and Good Clinical Practice guidelines [26].

### 2.3. Outcomes and Measures

Medical records were used to obtain basic sociodemographic and clinical data. The study used standardized tools to evaluate brittleness and adherence to recommendations.

The Tilburg Frailty Indicator (TFI) was developed by Gobbens et al. [27]. It consists of two parts: part A measures the health determinants of frailty syndrome, while part B includes 15 questions diagnosing the main components of frailty across three dimensions: physical (eight items), psychological (four items) and social (three items). For each subscale, there are no standards; however, interpretation of the individual frailty score can only be carried out by comparing it with the maximum score on a given subscale. Since each subscale contains more than one question, one can interpret results for each item. The maximum score is 15 points. The FS diagnosis is evidenced by obtaining at least 5 points [27]. The prefrail group used in the descriptive statistics contains patients who obtained 1–4 points. The study used a Polish adaptation of the questionnaire [28].

The Hill–Bone Compliance to High Blood Pressure Therapy Scales (HBCS) evaluates patient adherence to hypertension therapy. It consists of 14 questions forming three subscales: “Reduced sodium intake,” “Appointment-keeping,” “Taking antihypertensive drugs.” The respondent can choose one of six answers: “never,” “sometimes,” “often,” “constantly,” “not applicable,” “don’t know.” The subscale “Limiting sodium intake” contains three questions (with a score range of 3–12). The subscale “Going to doctor appointments” has two questions (with a score range of 2–8). The subscale “Taking medications” includes nine questions (with a score range of 9–36). The overall adherence score of this questionnaire ranges from 14 to 56 points with a higher score indicating more frequent violations of the rules of therapy. There are no standard scores for compliance with therapeutic recommendations. The study employed a Polish adaptation of questionnaire [29].

### 2.4. Statistical Analysis

Statistical analyses were conducted using SPSS v.24 (IBM SPSS Statistics, Chicago, IL, USA), Gretl and Python. Descriptive data were presented as frequencies of observations and percentages; mean, median, standard deviation (±SD), quartiles Q1–Q3, and minimum and maximum values were calculated. The normality of the data distribution was examined using the Kolmogorov–Smirnov test. Regression analysis tested the relationship between the Hill–Bone adherence subscales (dependent variables) and the Tilburg Frailty Indicator components (explanatory variables). Linear regression models were studied using residual analyses, Breusch–Pagan heteroskedasticity tests; the heteroskedasticity-corrected linear models were designed with Gretl software package. The Kruskal–Wallis tests were used to examine the relationship between and the Hill–Bone adherence subscales (dependent variables) and the categorical and ordered variables (gender, relationship status, education, salary, income professional activity, frailty classification, hypertension classification). A two-tailed *p*-value of *p* < 0.05 was considered statistically significant.

## 3. Results

Analysis showed that most of the respondents were women (52.9%), married or in a relationship (52.1%), received secondary education (46.8%), received annuity or pension (84.6%), earned a monthly income ≤ 1500 PLN/month (61.4%), had stage-2 hypertension based on the AHA (American Heart Association) classification (72.2%), and were diagnosed with diabetes (54.4%). Detailed data, including subgroups based on the frailty condition, are presented in Table 1.

Kruskal–Wallis analyses were performed (see Table 2) for the Hill–Bone “reduced sodium intake” scale. There was a significant difference between female and male participants (Me female = 4, Me male = 5, H = 11.3, *p* = 0.001).

For the Hill–Bone “appointment-keeping” score, there were significant differences in adherence by relationship status (Me single = 4, Me in a relationship = 3, H = 28.5 *p* < 0.001), education level (Me primary = 4, Me secondary = 4, Me higher = 3, H = 23,9, *p* < 0.001), salary (Me below 1501 PLN = 4, Me above 1500 PLN = 3, H = 13.5, *p* < 0.001), professional activity (Me active = 3, Me = retired = 4, Me unemployment = 3.5, H = 13.9, *p* = 0.001), and frailty (Me not frail = 4, Me prefrail = 3, Me frail = 4, H = 31.7, *p* < 0.001).

For the Hill–Bone “medication-taking” score, we found significant differences in adherence by relationship status (Me single = 13, Me in a relationship = 11, H = 38.3 *p* < 0.001), education level (Me primary = 13, Me secondary = 12, Me higher = 10, H = 33,4, *p* < 0.001), salary (Me below 1501 PLN = 13, Me above 1500 PLN = 3, H = 10.5, *p* < 0.001), professional activity (Me active = 11, Me = retired = 12, Me unemployment = 14.5, H = 11.4, *p* = 0.003), and frailty (Me not frail = 11, Me prefrail = 10, Me frail = 13, H = 68.5, *p* < 0.001).

Significant differences in overall Hill–Bone adherence were observed by relationship status (Me single = 22, Me in a relationship = 18, H = 47.8 *p* < 0.001), education level (Me primary = 22, Me secondary = 20, Me higher = 18, H = 35.7, *p* < 0.001), salary (Me below 1501 PLN = 21, Me above 1500 PLN = 18, H = 27.2, *p* = 0.001), professional activity (Me active = 19, Me retired = 20, Me unemployment 23, H = 9.7, *p* = 0.008), frailty (Me not frail = 19, Me prefrail = 18, Me frail = 21, H = 51.4, *p* < 0.001), and hypertension classification (Me normal = 25, Me elevated = 19, Me stage 1 = 19, Me stage 2 = 20, Me crisis = 19, H = 9.6, *p* = 0.048).

The TFI measurement showed that the mean frailty score was 7.09 (SD = 3.73). The most prominent frailty component concerned the physical domain (4.24 and SD = 2.54) (Table 3).

The Hill–Bone scale showed that the mean overall adherence was 20.51 (SD = 3.72) (Table 4).

Then, a linear regression analysis was performed to determine the relationships between HBCS domains (the dependent variables) and predictors based on the TFI. Additionally, the assumptions of specific regression models were examined based on a residue analysis. For models requiring data transformation, additional tables with corrected interdependency results of the studied variables are provided.

The linear regression model of the relationship between the Hill–Bone “reduced sodium intake” subscale and TFI domains is shown in Table 5A. The results indicated no dependency between the tested variables. Heteroskedasticity was confirmed for this model (*p* < 0.001). Accordingly, the analysis of the corrected regression model (see Table 5B) indicated no relationships between the variables; although there was a tendency for the “TFI—social” domain to demonstrate such a linkage (*p* = 0.098).

The regression analysis tested the relationship between the Hill–Bone “appointment-keeping” score and TFI domains. The significant predictors were variables of physical and social TFI domains (*p* = 0.002 and *p* < 0.0001, respectively) (Table 6). The model showed no homoskedasticity (*p* = 0.081).

The last regression model tested the relationship between the Hill–Bone “medication-taking” score and TFI domains. There were significant effects of the three TFI variables on adherence: physical (*p* < 0.0001), psychological (*p* = 0.003), and social (*p* < 0.0001) (see Table 7A). The analysis of heteroskedasticity indicated a significant result (*p* < 0.001). Then, the corrected regression model was built to examine the significance of the resulting associations. The analysis confirmed the significant relationships between the Hill–Bone “medication-taking” score and all TFI domains (*p* < 0.001 for each domain; R^2^ = 0.37) (Table 7B).

## 4. Discussion

Elderly patients with hypertension who develop frailty often show impaired physical, psychological, or social functioning. Frailty is associated with an 8-year mortality [30]. A meta-analysis by Vetrano et al. [31] showed that among patients with hypertension, 14% also have frailty syndrome, while frailty syndrome occurs in 46.5% of people over 65 with hypertension [32]. In fact, the most significant component of frailty syndrome turned out to be the physical component [28], which is consistent with the recent findings by Uchmanowicz et al. [33]. In contrast, the study by Chudiak et al. [34] suggested that for older hypertensive patients the social component of frailty played the most significant role, more likely resulting from poor fitness and diminished everyday life independence.

The treatment of hypertension requires patients to change their lifestyles (e.g., reducing salt intake) and take antihypertensive drugs [3]. To date, studies have shown that the median therapeutic adherence to antihypertensive therapy is 55.3% [35]. In the present study, the mean Hill–Bone adherence score was 20.51, which coincides with the results by Uchmanowicz et al. [33] and Chudiak et al. [34]. However, among hypertension patients living in Palestine the average score on the Hill–Hill–Bone scale was 24.9, which implies a lower adherence rate in comparison with the above studies [36].

Adherence to therapeutic recommendations may be influenced by demographic and socioeconomic factors, patient health status, and the operation of the health system, among other factors. Failure to adhere to recommendations makes it difficult to reduce the risk of adverse events and complications arising from untreated or improperly treated hypertension [19,37]. For example, the study by Abu-El-Noor [36] showed that the three most significant barriers reported by participants included “inability to exercise,” “inability to resist fast and fried food,” and “inability to keep themselves away from salty foods”.

The purpose of this work was to assess the relationship between frailty and compliance with therapeutic recommendations and cooperation in elderly patients with hypertension; only a few studies have attempted to analyze this dependence so far, and their results are inconclusive. A Brazilian study of hypertensive older adults [38] demonstrated no association between frailty and adherence to antihypertensive treatment. In contrast, the study of Jankowska-Polańska et al. [39] showed that frailty negatively affected adherence in elderly hypertensive patients, and lower adherence to the recommendations was diagnosed more often in frail patients than in nonfrail patients [40]. The study by Uchmanowicz et al. [33] showed a correlation between the frailty domains (physical, psychological, and social) and adherence to therapeutic recommendations in the aspect of “reduced sodium intake,” “appointment-keeping,” and “medication-taking.” In contrast, Chudiak et al. [34] demonstrated that TFI scores correlated with the global HBCS score (r = 0.509, *p* < 0.001) and two subscales: “appointment-keeping” (r = 0.34, *p* < 0.001) and “medication-taking” (r = 0.537, *p* < 0.001).

This study confirmed that frailty syndrome and its components are factors affecting adjustment to therapeutic recommendation. The results showed that the physical and social domains of frailty affected adherence to therapeutic recommendations in patients with hypertension in the dimensions of “appointment-keeping” and “medication-taking.” Possibly, this result may be due to reduced interpersonal contacts in aged patients, which may limit the possibility for their assistance, and decreased functional fitness limiting numbers of visits to specialists. Additionally, the psychological component may affect adherence to therapeutic recommendations in patients with hypertension by influencing medication-taking behaviors.

Aging progressively deteriorates the function of all systems and outcomes of the human body, including its cognitive functions. Additionally, when elderly individuals experience depression symptoms, poorer cognitive functioning may be observed [41]. Reduced cognitive functioning within domains of perception, memory, and attention can produce non-adherent behavior (e.g., forgetting to take medications regularly).

This study has some limitations. First, the analysis of adherence to therapeutic recommendations was based only on the questionnaire data. Secondly, the patients were hospitalized for exacerbation of hypertension symptoms, suggesting the non-compliance with the recommendations as their possible originate. Therefore, the obtained results should be carefully generalized for patients with hypertension.

## 5. Conclusions

The diagnosis of frailty syndrome can negatively influence adherence to therapeutic recommendations in patients with hypertension.

### Practical Implications

Knowledge of frailty syndrome and its evaluation has become a contemporary requirement for members of the therapeutic team who face difficulties in making effective diagnosis and treatment of the hypertension condition among the elderly. Frailty is an independent factor influencing adherence to therapeutic recommendations by patients with hypertension. Therefore, special attention should be given to patients with this syndrome to prevent the negative consequences of untreated hypertension and the risk of adverse outcomes, i.e., premature mortality or sudden cardiovascular incidents. Systematic measures of FS should be implemented to enable quick diagnosis of patients predisposed to the development of frailty. Frailty interventions include exercise, nutritional supplementation, psychosocial support, drug management, and comprehensive assessment, but a systematic management standard is still lacking. Eventually, advancing clinical and nursing knowledge about the relationship between frailty and adherence to recommendations in elderly patients with hypertension will increase their quality of life [42].

## Figures and Tables

**Table 1:** Demographic and clinical characteristics of the total group of HBP patients (*n* = 259).

Variables	Non-Frail (TFI = 0, *n* = 5)	Prefrail (TFI = 1:4, *n* = 78)	Frail (TFI = 5:15, *n* = 176)	*n* = 259 (%) *
Gender				
Male	3 (60.0)	43 (55.1)	76 (43.2)	122 (47.1)
Female	2 (40.0)	35 (44.9)	100 (56.8)	137 (52.9)
Age (years)	65.00 ± 0.00	67.14 ± 4.35	73.56 ± 8.29	71.46 ± 7.86
BMI (kg/m^2^)	24.97 ± 1.91	28.19 ± 5.08	26.27 ± 14.96	26.82 ± 12.67
Relationship status				
Married/in relationship	4 (80.0)	63 (80.8)	68 (38.6)	135 (52.1)
Single	1 (20.0)	15 (19.2)	108 (61.4)	124 (47.9)
Education level				
Primary school or lower	0 (0.0)	8 (10.3)	61 (34.7)	69 (26.6)
Secondary education	2 (40.0)	36 (46.2)	83 (47.2)	121 (46.8)
Higher education	3 (60.0)	34 (43.6)	32 (18.2)	69 (26.6)
Income				
<1500 PLN/month	1 (20.0)	32 (41.0)	126 (71.7)	159 (61.4)
>1500 PLN/month	4 (80.0)	46 (59.0)	50 (28.3)	100 (38.6)
Professional activity				
Active	2 (40.0)	19 (24.4)	17 (9.7)	38 (14.7)
Retirement / pension	3 (60.0)	58 (74.4)	158 (89.7)	219 (84.6)
Unemployed	0 (0.0)	1 (1.3)	1 (0.6)	2 (0.8)
Years since HT diagnosis	9.60 ± 5.41	10.06 ± 7.04	15.20 ± 8.57	13.54 ± 8.42
Level of hypertension (AHA standards)				
Normal	0 (0.0)	1 (1.3)	12 (6.8)	13 (5)
Elevated	0 (0.0)	1 (1.3)	5 (2.8)	6 (2.3)
Stage 1	2 (40.0)	18 (23.1)	27 (15.3)	47 (18.1)
Stage 2	2 (40.0)	56 (71.8)	129 (73.3)	187 (72.2)
Crisis	1 (20.0)	2 (0.8)	3 (1.7)	6 (2.3)
Other chronic diseases				
None	0 (0.0)	21 (26.9)	15 (8.5)	36 (13.9)
Diabetes	3 (60.0)	36 (46.2)	102 (58.0)	141 (54.4)
Hypercholesterolemia	2 (40.0)	21 (26.9)	59 (33.5)	82 (31.7)
Coronary artery disease (CAD)	1 (20.0)	10 (12.8)	60 (34.1)	71 (27.4)
Kidney failure	0 (0.0)	6 (7.7)	23 (13.1)	29 (11.2)
Rheumatic diseases	0 (0.0)	10 (12.8)	37 (21.0)	47 (18.1)

* Average and standard deviations for age, years since HT diagnosis and BMI.

**Table 2:** Differences in Hill—Bone subscales in HBP patients given clinical and sociodemographic data.

		Adherence Scales and Subscales (Medians, H and *p*-Values)							
		Reduced Sodium Intake		Appointment Keeping		Medication Taking		Total	
Sex	Female (*n* = 137)	4	H = 11.3 (*p* = 0.001)	4	H = 1.9 (*p* = 0.165)	12	H = 0.1 (*p* = 0.784)	20	H = 0.3 (*p* = 0.557)
	Male (*n* = 122)	5		3		11		20	
Relationship status	Single (*n* = 124)	5	H = 0.5 (*p* = 0.482)	4	H = 28.5 (*p* < 0.001)	13	H = 38.3 (*p* < 0.001)	22	H = 47.8 (*p* < 0.001)
	Not single (*n* = 135)	5		3		11		18	
Education	Primary school or lower (*n* = 69)	5	H = 1.3 (*p* = 0.533)	4	H = 23.9 (*p* < 0.001)	13	H = 33.4 (*p* < 0.001)	22	H = 35.7 (*p* < 0.001)
	Secondary education (*n* = 121)	5		4		12		20	
	Higher education (*n* = 69)	5		3		10		18	
Income	<1500 PLN (*n* = 159)	5	H = 0.0 (*p* = 0.840)	4	H = 13.5 (*p* < 0.001)	13	H = 29.9 (*p* < 0.001)	21	H = 27.2 (*p* < 0.001)
	≥1500 PLN (*n* = 100)	5		3		10.5		18	
Professional Activity	Active (*n* = 38)	5	H = 5.7 (*p* = 0.058)	3	H = 13.9 (*p* = 0.001)	11	H = 11.4 (*p* = 0.003)	19	H = 9.7 (*p* = 0.008)
	Retirement/pension (*n* = 219)	5		4		12		20	
	unemployed (*n* = 2)	5		3.5		14.5		23	
Frailty	Not frail (*n* = 5)	4	H = 2.7 (*p* = 0.262)	4	H = 31.7 (*p* < 0.001)	11	H = 68.5 (*p* < 0.001)	19	H = 51.4 (*p* < 0.001)
	Prefrail (*n* = 78)	5		3		10		18	
	Frail (*n* = 176)	5		4		13		21	
Hypertension	Normal (*n* = 13)	5	H = 5.2 (*p* = 0.268)	4	H = 2.8 (*p* = 0.590)	16	H = 9.1 (*p* = 0.060)	25	H = 9.6 (*p* = 0.048)
	Elevated (*n* = 6)	5		3		11		19	
	Stage 1 (*n* = 47)	4		3		11		19	
	Stage 2 (*n* = 187)	5		3		12		20	
	Crisis (*n* = 6)	4.5		3.5		10		19	

**Table 3:** Assessment of physical, psychological, and social domains by the Tilburg Frailty Indicator (TFI) in HBP patients.

TFI Domains	*n*	M	SD	Me	Q1	Q3	Min	Max
TFI—physical	259	4.24	2.54	4.00	2.00	7.00	0.00	8.00
TFI—psychological	259	1.75	1.02	2.00	1.00	2.00	0.00	4.00
TFI—social	259	1.09	0.92	1.00	0.00	2.00	0.00	3.00
Total TFI	259	7.09	3.73	8.00	4.00	10.00	0.00	15.00

**Table 4:** Assessment of three behavioral domains by the Hill–Bone Compliance to High Blood Pressure Therapy Scale in HBP patients.

Domain	*n*	M	±SD	Me	Q1	Q3	Min	Max
Diet-reduced sodium intake	259	4.73	1.14	5.00	4.00	5.00	3.00	12.00
Appointment-keeping	259	3.44	1.05	3.00	3.00	4.00	2.00	7.00
Medication-taking	259	12.33	2.89	12.00	10.00	14.00	9.00	23.00
Total score	259	20.51	3.72	20.00	18.00	23.00	14.00	33.00

**Table 5:** (A) Linear regression analysis with the Hill–Bone “reduced sodium intake” variable as the dependent variable. (B) Heteroskedasticity-corrected model assessing the TFI impact on the Hill–Bone “reduced sodium intake” score.

A						
Model	Unstandardized Coefficients		Standardized Coefficient	*t*-Ratio	*p*-Value	R^2^
	B	SE	β			
constant	4.98	0.15		32.30	<0.0001	0.02
TFI—physical	−0.03	0.04	−0.08	−0.96	0.338	
TFI—psychological	−0.13	0.09	−0.12	−1.49	0.138	
TFI—social	0.11	0.09	0.09	1.34	0.182	
**B**						
**Model: Heteroskedasticity-Corrected, Using Observations *n* = 259** **Dependent Variable: Hill–Bone “Reduced-Sodium-Intake” Score**						
	**Coefficient**		**SE**	**t-Ratio**	***p*-Value**	**R^2^**
constant	5.03		0.16	30.67	<0.0001	0.02
TFI—physical	−0.06		0.04	−1.59	0.113	
TFI—psychological	−0.12		0.08	−1.43	0.153	
TFI—social	0.14		0.08	1.66	0.098 **	

**: tendency to be statistically significant.

**Table 6:** Linear regression analysis with the “appointment-keeping” domain as the dependent variable.

Model	Unstandardized Coefficients		Standardized Coefficient	*t*-Ratio	*p*-Value	R^2^
	B	SE	β			
Constant	2.72	0.13		20.84	<0.0001 *	0.17
TFI—physical	0.10	0.03	0.23	3.20	0.002 *	
TFI—psychological	−0.02	0.07	−0.02	−0.24	0.814	
TFI—social	0.32	0.07	0.28	4.38	<0.0001 *	

*: statistically significant.

**Table 7:** (A) Linear regression analysis with the “medication-taking” domain as the dependent variable. (B) Heteroskedasticity-corrected model testing the TFI impact on the Hill–Bone “medication-taking” score.

A						
Model	Unstandardized 259 Coefficients		Standardized Coefficient	*t*-Ratio	*p*-Value	R^2^
	B	SE	Β			
constant	9.16	0.33		28.18	<0.0001	0.33
TFI—physical	0.36	0.08	0.32	4.82	<0.0001 *	
TFI—psychological	0.54	0.18	0.19	2.96	0.003 *	
TFI—social	0.63	0.18	0.20	3.54	<0.0001 *	
**B**						
**Model: Heteroskedasticity-Corrected, Using Observations *n* = 259** **Dependent Variable: “Medication-Taking”**						
	**Coefficient**		**SE**	***t*-Ratio**	***p*-Value**	**R^2^**
constant	9.43		0.19	48.79	<0.0001	0.37
TFI—physical	0.28		0.07	4.24	<0.0001 *	
TFI—psychological	0.47		0.15	3.23	0.001 *	
TFI—social	0.76		0.16	4.87	<0.0001 *	

*: statistically significant.

## Data Availability

The data presented in this study are available on request from the corresponding author (A.K.).
